# True Knot of the Umbilical Cord and Associated Adverse Perinatal Outcomes: A Case Series

**DOI:** 10.7759/cureus.35377

**Published:** 2023-02-23

**Authors:** Vidya Gaikwad, Suneha Yalla, Pankaj Salvi

**Affiliations:** 1 Obstetrics and Gynecology, Dr. D. Y. Patil Medical College, Hospital and Research Centre, Dr. D. Y. Patil Vidyapeeth (Deemed to be University), Pune, IND

**Keywords:** stillbirth, iufd, perinatal asphyxia, umbilical cord, true knot

## Abstract

An actual knot that forms during pregnancy is known as a true knot of the umbilical cord (TKUC) which is seen in 0.3% to 1.2% of pregnancies. TKUC is noteworthy because it can lead to a variety of adverse perinatal outcomes, including infants with low Apgar scores, small for gestational age (SGA) fetuses, fetal hypoxia, and also in some cases fetal death. Here, we present instances of TKUC of three patients and the various associated perinatal outcomes.

## Introduction

The term "true knot of the umbilical cord" (TKUC) refers to a knot that actually forms during pregnancy, while the term "false knot" describes a bulge in the cord caused by excessive Wharton's jelly or looping of the cord vessels [1]. True umbilical cord knots are important because they can result in a variety of negative perinatal outcomes, including small for gestational age (SGA) fetuses, low Apgar scores at birth, fetal hypoxia and even fetal death [2,3].

Since the fetal blood supply is derived from the umbilical cord, any defect in the cord may significantly affect the fetal outcome. Numerous factors including increased cord length, polyhydramnios, male babies, amniocentesis, monoamniotic twins, grand multiparity and tiny fetuses can predispose to TKUC. The risk of fetal death from TKUC is up to four times higher than that from other causes, and it can have a wide range of adverse effects on pregnancy and labour [4].

## Case presentation

Case 1

A 27-year-old primigravida with gestational diabetes mellitus (GDM) at term gestation presented with labour pain, which was not associated with leaking or bleeding per vaginum. She was a known case of GDM on medical nutrition therapy (MNT) diagnosed at 28 weeks of gestation.

**Figure 1:**
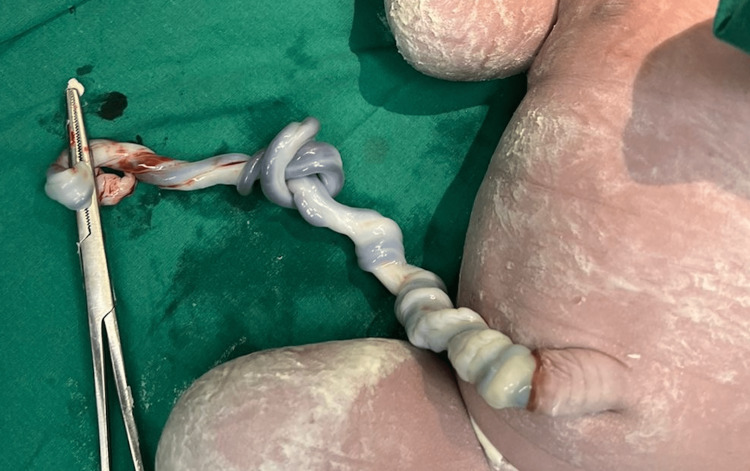
One true tight knot in the umbilical cord of deceased fetus.

At the time of presentation, her vitals were stable and blood sugars were well controlled. On abdominal examination, mild uterine contractions were present and fetal heart rate was 150 bpm. Patient was in latent labour. Ultrasonography (USG) done on admission showed a single live fetus corresponding to 37+1 weeks of gestation with estimated fetal weight (EFW) of 3.2 kg, amniotic fluid index (AFI) of 16 cm and normal colour Doppler. Non-stress test (NST) was reactive. Labour was allowed to progress spontaneously with fetal heart rate (FHR) monitoring. Six hours later, there was sudden disappearance of fetal heart sounds (FHS) on cardiotocogram (CTG). Intrauterine fetal demise (IUFD) was confirmed on ultrasound. Labour was augmented and she delivered a male fetus weighing 3 kg, five hours later. One true tight knot (Figure 1) was noted in the long umbilical cord measuring 70 cm.

Case 2

A 31-year-old second gravida with previous caesarean section presented at 33 weeks of gestation with USG suggestive of fetal growth restriction (FGR), oligohydramnios and uteroplacental insufficiency. Patient complained of decreased fetal movements since two days. Antenatal period of the patient was uneventful.

Her vitals were stable on general examination. On per abdominal examination, fundal height was found to correspond with 30 weeks gestation. USG showed EFW of 1339 g (<5th centile) and AFI of 3-4 cm (oligohydramnios), mean uterine artery (pulsatility index, PI) >95th centile suggestive of uteroplacental insufficiency.

**Figure 2:**
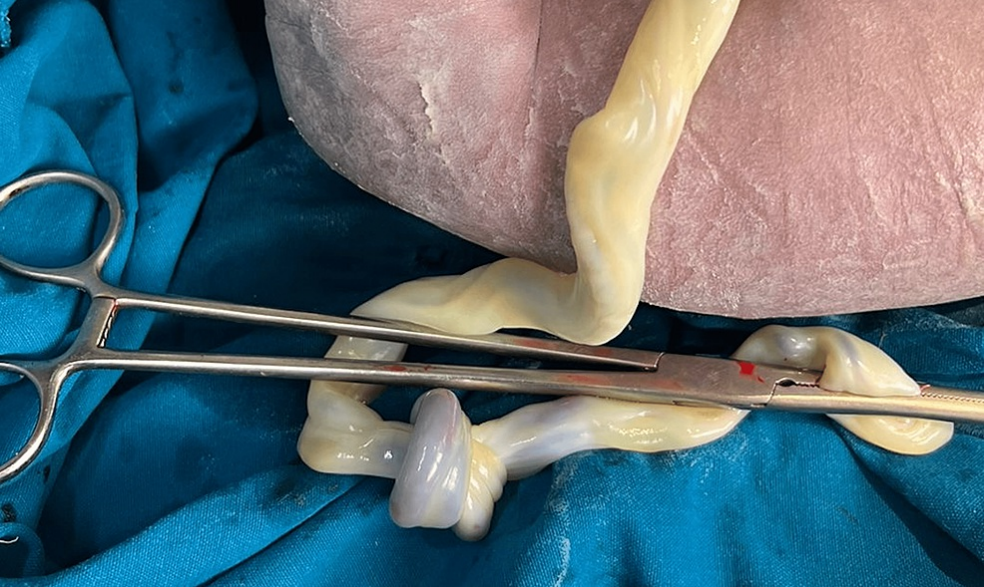
Cord of preterm fetus showing true knot.

Emergency lower segment cesarean section in view of oligohydramnios, FGR and previous caesarean section was performed. A male baby weighing 1350 g was delivered. The fetus had a single loop of cord around neck with a true tight knot (Figure 2). Due to low birth weight and prematurity, the baby was admitted in the neonatal intensive care unit (NICU) with an Apgar score of 5 at one minute after birth. After 15 days of NICU stay, the baby was discharged.

Case 3

**Figure 3:**
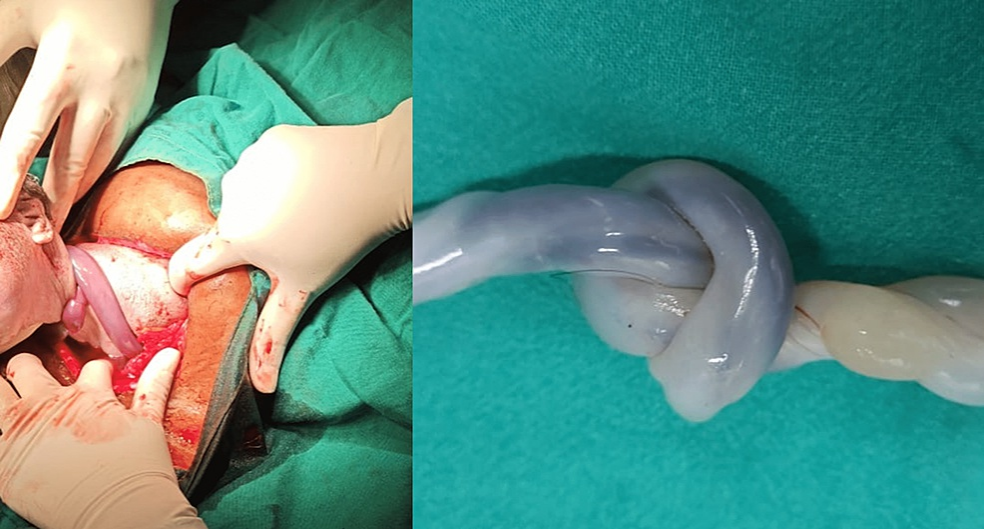
Preterm deceased fetus with loop of cord around neck, with true knot of umbilical cord.

A 32-year-old multigravida who had two prior caesarean deliveries presented at a gestational age of 32 weeks with a USG that suggested IUFD. When she first presented, her vitals were stable and abdominal examination revealed that her fundal height matched a 32-week pregnancy and uterus was relaxed with no scar tenderness. FHS could not be localised. A single intrauterine dead fetus at 30 weeks of gestation with one loop of cord around its neck and an EFW of 1600 g and an AFI of 12 cm was identified by ultrasonography. At caesarean section, a female child weighing 2100 g was delivered. The fetus had a loop of cord around the neck with a true tight knot (Figure 3).

## Discussion

Since the umbilical cord is from where the fetus gets its blood supply, any defect therein may have a profound impact on the fetal fate. Predictions state that TKUC frequently manifests between nine and 12 weeks of pregnancy, when sufficient liquor and vigorous fetal movements allow the fetus to pass through a cord loop [4]. The majority of difficulties, however, take place in the later stages of pregnancy when the fetal blood supply is affected by the cord's tightening in response to fetal movements and the decreased liquor volume with increasing gestation. Numerous conditions, including polyhydramnios, increased cord length, monoamniotic twins, male babies, grand multiparity, small fetuses and amniocentesis can predispose to TKUC [5].

Notably, in our case, two of the three women gave birth to male babies and two of them were multigravidas. For two of them, the cord length fell within the normal range. Male fetuses are thought to be more active, which results in the development of TKUC. TKUC can result in a number of adverse pregnancy and labor outcomes, including an SGA fetus, a low Apgar score at birth, fetal hypoxia, and fetal death [3]. The risk of fetal death is up to four times higher in TKUC [6-8]. One case of sudden IUFD in a patient with controlled GDM on MNT, one case of FGR with oligohydramnios and one case of IUFD due to TKUC were discussed in this case series.

The four-leaf clover or the "hanging noose sign" is a distinctive multicolor pattern in the cord that can be used to identify TKUC antenatally due to the development of cutting-edge technology like three-dimensional/four-dimensional colour Doppler ultrasound [2]. Only 12% of prenatal cases of TKUC are detected [3]. It largely goes unnoticed until it is visually observable in real time [4].

Prenatal diagnosis of TKUC has been a chance finding and the true effect of this condition on neonatal outcomes has not yet been assessed. However, a true umbilical cord knot detected during pregnancy should prompt additional monitoring of the pregnancy and Doppler flow patterns [9]. The detection of an umbilical cord true knot during pregnancy is challenging with 2D ultrasound however 3D and Doppler ultrasound are now readily available. It is important to distinguish between true and false knots [3].

The true umbilical cord knot may cause unrecognized morphological and circulatory symptoms, resulting in acute hypoxia, as indicated by an increased umbilical-cerebral Doppler ratio. Factors, yet unknown, contribute to antenatal tightening of a true knot. Some hypotheses state that the fetus itself tightens the knot by either tangling its feet around the cord or possibly by clutching the cord with a hand [1].

Review of current literature supports the following points: 1. The majority of findings of true knot in the umbilical cord remain incidental despite advancements in prenatal sonography and are usually without adverse perinatal outcomes. Therefore, many true knots may go undiagnosed despite prenatal sonographic screening, leading one to conclude that true knots are not always amenable to prenatal diagnosis and are not always linked to poor perinatal outcomes [10]. 2. Percentage of TKUC linked to an increased risk of perinatal morbidity and stillbirth is yet to be determined [10]. 3. Prenatal sonography is unable to identify cases of true umbilical cord knots that will eventually tighten or alternatively, be linked to a poor perinatal outcome, such as stillbirth [10]. 4. When a true umbilical cord knot is identified, vaginal delivery at term is not contraindicated. In the absence of non-reassuring fetal status or Doppler velocimetry changes revealing increased downstream resistance to flow in the umbilical artery proximal to the knot, indicating possibility of umbilical cord compression, caesarean delivery is unwarranted for the diagnosis of a TKUC [10].

Given the possibility of a negative perinatal outcome in the case of true knots of the umbilical cord, it seems that the documentation and an open discussion with patients with gestational age above viability, regarding daily fetal movement assessment, and interval fetal testing until delivery, should be taken into consideration or carried out, although regrettably it is evident that stillbirth cannot be prevented in the interim between diagnosis and delivery. An effective and viable clinical care strategy is to maintain expectant management with interval fetal testing while watching for the onset of spontaneous labour [10].

Although there is no specific management for these cases [11], TKUC can be detected early in pregnancy and closely watched until fetal maturity is reached [12].

## Conclusions

Although TKUC is a common disorder with serious perinatal outcomes, it is still not understood how significant a prenatal diagnosis of the condition is. In otherwise low-risk pregnancies, TKUC should be suspected in patients with risk characteristics and attention should be placed on its antenatal detection on ultrasonography. This will help to minimise unfavourable fetal outcomes and obstetric disasters.

Notwithstanding in our assessment, patients with a true knot in the umbilical cord identified by prenatal sonography and fetuses that are vertex-presenting at or >37 weeks of gestation should consider delivery as a possible option in order to prevent stillbirth. Although a caesarean delivery is clearly contraindicated for individuals with a true umbilical cord knot identified by prenatal sonography, the patient ultimately has the final say in this matter, otherwise constant intrapartum electronic fetal monitoring is necessary.
